# Pan-cancer biomarkers of immune therapy resistance: mechanisms and clinical translation

**DOI:** 10.3389/fcell.2026.1895670

**Published:** 2026-10-07

**Authors:** Chao Wang, Dong Yang

**Affiliations:** Department of Gastric Surgery, Liaoning Cancer Hospital & Institute, Shenyang, China

**Keywords:** biomarkers, immune therapy resistance, mechanisms, pan-cancer, clinical translation

## Abstract

Immune checkpoint blockade targeting PD-1/PD-L1 has reshaped cancer therapy across multiple tumor types. However, primary and acquired resistance remain major barriers to durable clinical benefit. Increasing evidence indicates that immunotherapy resistance is not driven by isolated molecular alterations, but by coordinated changes involving tumor-intrinsic immune escape, immunosuppressive tumor microenvironments, systemic inflammatory remodeling, and adaptive biomarker evolution under therapeutic pressure. This review focuses on pan-cancer biomarkers of immune therapy resistance from the perspective of mechanisms and clinical translation. First, we summarize targetable biomarker classes that link resistance biology to therapeutic sensitization across cancers, including transcription factors, genomic alterations, immune checkpoint proteins and metabolic regulators. For each class, we highlight representative cancer types in which experimental or clinical evidence supports their role in immunotherapy resistance. Second, we discuss the mechanistic basis of resistance-associated biomarkers, emphasizing antigen presentation defects, impaired interferon signaling, oncogenic pathway activation, T-cell exhaustion, myeloid and stromal immunosuppression, metabolic reprogramming, and dynamic systemic immune remodeling. These mechanisms provide a biological perspective for interpreting biomarkers not only as predictors of treatment response, but also as functional indicators of resistance evolution. By integrating biomarker classification, resistance mechanisms, and therapeutic sensitization strategies, this review aims to provide a new perspective for using pan-cancer resistance biomarkers to guide rational combination immunotherapy and improve precision cancer treatment.

## 1 Introduction

In recent years, cancer immunotherapy, particularly immune checkpoint inhibitors, has profoundly reshaped the treatment landscape across multiple malignancies. Among these strategies, therapies targeting PD-1/PD-L1 have demonstrated durable antitumor activity in a wide range of cancers, including melanoma (Robert et al., 2019), non-small cell lung cancer (NSCLC) (Reck et al., 2016), renal cell carcinoma (RCC), head and neck squamous cell carcinoma (HNSCC) (Harrington et al., 2023), gastric cancer (Janjigian et al., 2021), and colorectal cancer (André et al., 2020). Accordingly, PD-1/PD-L1 blockade has become an increasingly important component of systemic treatment for advanced cancers. Despite these advances, only a subset of patients achieve long-term survival benefits from immunotherapy. A considerable proportion of patients exhibit primary non-response, while others eventually develop acquired resistance after an initial clinical response. Immunotherapy resistance arises from complex and interrelated biological processes, including tumor-intrinsic immune escape, impaired antigen presentation, T-cell exhaustion, the establishment of an immunosuppressive tumor microenvironment, and dysregulated metabolic and inflammatory networks. These mechanisms represent major barriers to achieving clinical benefit from immunotherapy; therefore, identifying immunotherapy-responsive patient populations is of considerable clinical significance.

Notable progress has been made in identifying biomarkers associated with immunotherapy response and resistance. Recent study had highlighted the critical role of biomarkers in predicting responses to immunotherapy, identifying potential beneficiary populations, and guiding precision immunotherapy (Subbiah et al., 2024). Furthermore, emerging evidence suggests that certain resistant states may be therapeutically modifiable. Study had shown that CTLA-4 blockade may improve treatment efficacy in patients with metastatic NSCLC harboring STK11 and KEAP1 mutations among those who exhibit poor responses to PD-L1 targeted therapy (Skoulidis et al., 2024). Previous reviews have primarily focused on resistance to a single class of drugs or a specific treatment regimen in a particular tumor type, such as resistance to tyrosine kinase inhibitors in EGFR-mutant NSCLC, endocrine therapy resistance in hormone receptor-positive breast cancer, gemcitabine resistance in pancreatic cancer, and resistance to targeted therapies in BRAF V600-Mutated metastatic melanoma (Zhou et al., 2025; Hank et al., 2020; Koltai et al., 2022; Florent et al., 2023). Evidence remains fragmented across biomarker classes, tumor types, detection methods, resistance mechanisms, and levels of clinical validation, making it difficult to identify biomarkers that are sufficiently robust and actionable for clinical application. Together, the evidence summarized in these studies suggests that resistance biomarkers may serve not only as predictors of treatment failure but also as indicators of biologically distinct and potentially actionable resistance states.

To systematically elucidate potential biomarkers of immunotherapy response and the underlying mechanisms of therapeutic resistance in solid tumors, this review systematically categorizes resistance-associated mechanisms and potential biomarkers into four molecular levels: transcription factors and transcriptional regulators, gene mutations and genomic alterations, proteins and immune checkpoint molecules, and metabolites and metabolic pathway–related biomarkers. Each category is further mapped to its corresponding mechanisms of therapeutic resistance. Beyond identifying biomarker–response associations, we evaluate their clinical utility based on evidence strength, cross-cancer consistency, clinical feasibility, and availability of matched therapies. By integrating biomarker characteristics with their underlying mechanisms, clinical evidence, and therapeutic implications, this review seeks to distinguish resistance biomarkers with meaningful clinical potential from those that remain insufficiently validated, thereby informing more individualized combination immunotherapy strategies.

## 2 Targetable molecular determinants and biomarkers linking immunotherapy resistance to therapeutic sensitization across cancers

Immunotherapy resistance is driven by diverse tumor-intrinsic and microenvironmental mechanisms that impair antigen presentation, disrupt immune signaling, suppress effector-cell activity, or establish metabolically unfavorable conditions. Biomarkers reflecting these processes are therefore valuable not only for identifying primary or acquired resistance, but also for revealing therapeutically actionable vulnerabilities that may restore or enhance responsiveness to immune checkpoint blockade. According to the biological level at which these resistance mechanisms operate, targetable biomarkers can be broadly classified into four major categories: transcription factor-related biomarkers, genomic alteration-related biomarkers, protein and immune checkpoint-related biomarkers, and metabolite or metabolism-related pathway biomarkers. Together, these classes provide a perspective for linking the molecular basis of immune escape with potential sensitization strategies across cancer types, while also distinguishing targets supported primarily by preclinical evidence from those that have already demonstrated clinical activity (Figure 1).

**FIGURE 1:**
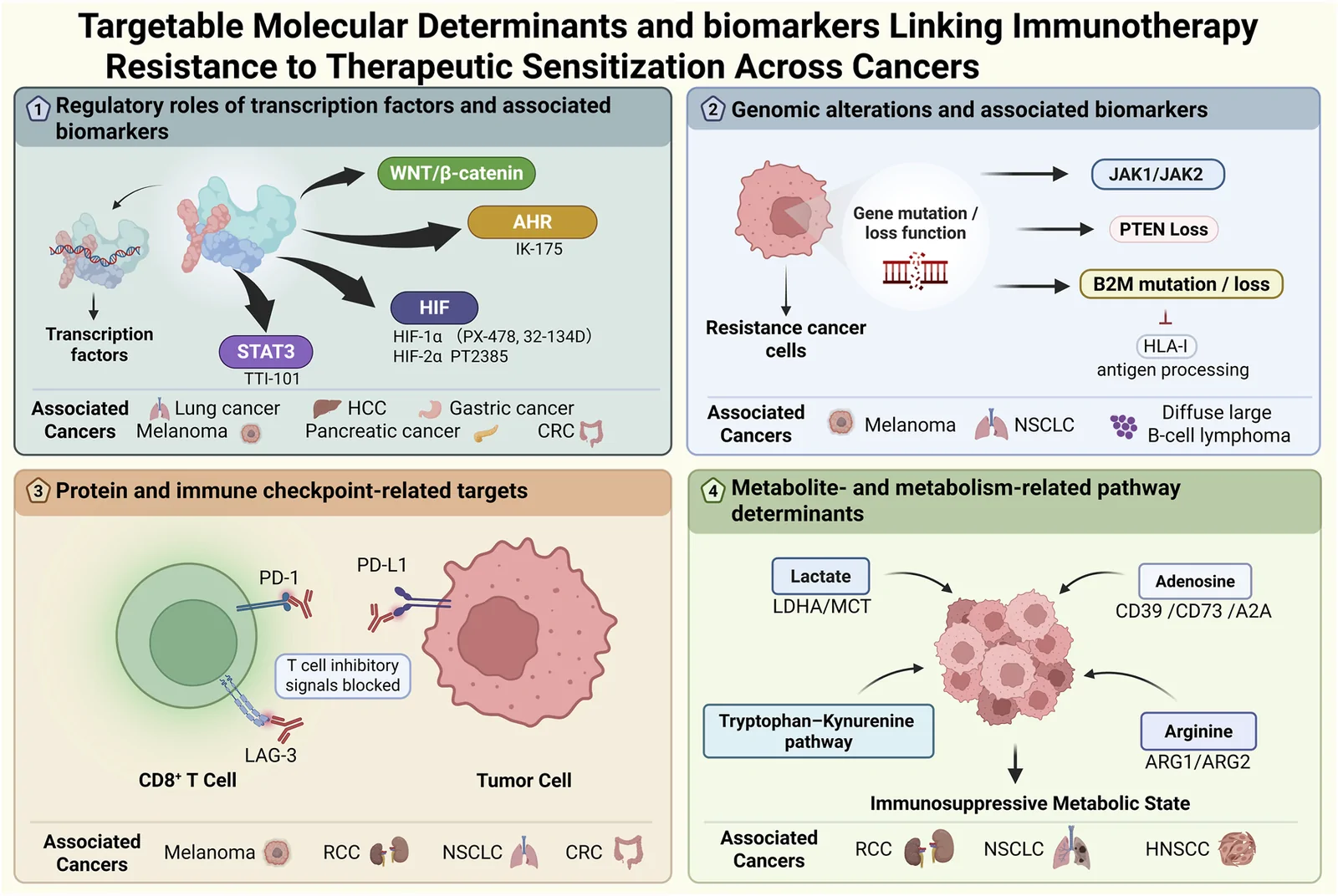
Classification of clinically relevant and targetable biomarker categories associated with immunotherapy resistance and therapeutic sensitization across multiple cancer types.

### 2.1 Regulatory roles of transcription factors and associated biomarkers

The activation status of immune-related or resistance-associated pathways is often reflected by the activation and nuclear translocation of transcription factors or transcriptional co-regulators, which regulate gene-expression programs governing tumor immunity and therapeutic resistance. Preclinical studies have demonstrated that STAT3 blockade may enhance antitumor immune responses in lung cancer, gastric cancer, hepatocellular carcinoma, pancreatic cancer, and melanoma. Moreover, orally available STAT3 inhibitors, including TTI-101, have entered Phase I clinical trials for multiple solid tumors (Clowers et al., 2025; Tsimberidou et al., 2025; Sun et al., 2013; Zhu et al., 2026; Datta et al., 2022; Huang et al., 2021). In the Phase I study of TTI-101, paired on-treatment tumor biopsies from patients with high baseline pY-STAT3 levels showed a reduction in pY-STAT3, supporting its use as a pharmacodynamic biomarker of target engagement (Tsimberidou et al., 2025). HIF blockade has demonstrated the potential to enhance immunotherapy across several malignancies. Current studies have demonstrated that targeted inhibition of HIF can reverse immune effector dysfunction and improve responses to immunotherapy. These approaches include HIF-1α inhibitors such as PX-478 and 32–134D, as well as the HIF-2α inhibitor PT2385 (Choueiri et al., 2026; Salman et al., 2022; Luo et al., 2022; Ma et al., 2022; Espinoza et al., 2025). In a clinical translational study of PT2385, decreased circulating erythropoietin reflected systemic HIF-2 inhibition, while dissociation of the HIF-2α/HIF-1β complex and suppression of HIF-2 target-gene expression in paired tumor biopsies provided more direct evidence of intratumoral target engagement. Conversely, the HIF-2α G323 E gatekeeper mutation impaired PT2385 binding, maintained HIF-2 complex formation and transcriptional activity during treatment, and may serve as a candidate biomarker of resistance (Courtney et al., 2020). The aryl hydrocarbon receptor (AHR) is an important regulator of tumor-associated immunosuppression, and its aberrant activation has been implicated in adaptive resistance following PD-1 blockade. Recent clinical studies demonstrated that the oral AHR inhibitor IK-175 exhibited favorable safety and preliminary antitumor activity in patients with advanced solid tumors refractory to prior anti-PD-1 therapy (Aggen et al., 2026; McGovern et al., 2022). High nuclear AHR expression may serve as a candidate enrichment or potential predictive biomarker for IK-175 therapy, whereas treatment-induced modulation of AHR target genes, including CYP1A1 and CYP1B1, together with inhibition in *ex vivo* AHR activation assays, may serve as pharmacodynamic markers of target engagement (Aggen et al., 2026; McGovern et al., 2022). In addition to classical transcription factors such as AHR and HIF, the transcription-regulating WNT/β-catenin signaling axis has recently emerged as an important regulator of the tumor immune microenvironment. Through β-catenin-mediated transcriptional activation, this pathway drives immune exclusion and promotes resistance to immune checkpoint inhibition. Studies have demonstrated that the WNT/β-catenin signaling pathway plays an important role in tumor immune evasion and resistance to immune checkpoint inhibitors. Persistent activation of this pathway can induce T-cell exclusion and promote an immunosuppressive tumor microenvironment. Targeted inhibition of the WNT/β-catenin signaling pathway may reverse immunosuppressive states and enhance sensitivity to immunotherapy in multiple solid tumors, including melanoma, hepatocellular carcinoma, colorectal cancer, and non-small cell lung cancer (DeVito et al., 2021; Wu et al., 2025; Wang et al., 2020; Takeuchi et al., 2021). Tumor-intrinsic WNT/β-catenin pathway activation, as indicated by β-catenin signaling elements (CTNNB1, APC, AXIN) alterations, nuclear β-catenin accumulation, or a WNT/β-catenin transcriptional signature, may serve as a candidate biomarker of immune exclusion and primary resistance to immune checkpoint inhibitors, with potential utility for identifying patients less likely to benefit from immunotherapy and guiding treatment stratification (Luke et al., 2019; Mut et al., 2025).

### 2.2 Genomic alterations and associated biomarkers

Tumor-intrinsic genomic alterations may drive the development of primary or acquired resistance to immunotherapy. B2M mutations or loss impair HLA-I complex stability and surface HLA-I expression, thereby disrupting tumor antigen presentation and preventing effective recognition and killing of tumor cells by CD8^+^ T cells. Previous studies have shown that, in melanoma, lung cancer, and diffuse large B-cell lymphoma, B2M mutation or loss can disrupt HLA-I-mediated antigen processing and presentation, thereby enabling tumor immune escape (Zaretsky et al., 2016; Gettinger et al., 2017; Challa-Malladi et al., 2011). From a biomarker perspective, B2M loss-of-function mutations or deletions can be detected by genomic sequencing, whereas B2M and HLA-I protein expression can be assessed by immunohistochemistry. Compared with the detection of genomic alterations alone, concurrent loss of B2M expression and cell-surface HLA-I expression provides stronger evidence of impaired antigen-presentation capacity. Therefore, combined B2M/HLA-I loss may serve as a candidate biomarker of resistance to T-cell-dependent immunotherapies. JAK1/2 loss-of-function mutations mediate primary resistance to PD-1 blockade by disrupting IFN-γ/JAK-STAT signaling, resulting in impaired PD-L1 induction, defective antigen presentation, and reduced T-cell recruitment, thereby enabling tumor immune evasion (Shin et al., 2017; Shen et al., 2017). MSI-H status represents an important determinant for predicting response to immune checkpoint blockade. Although some MSI-H tumors harbor B2M or JAK1/2 loss-of-function mutations that impair antigen presentation or IFN-γ signaling, these patients may still benefit from PD-1 blockade, suggesting that the high mutational burden and immunogenicity of MSI-H tumors can overcome certain immune escape mechanisms (Zhang et al., 2022; Middha et al., 2019). PTEN loss promotes immune escape and resistance to immunotherapy across multiple tumor types, including melanoma and lung cancer. Conversely, exogenous PTEN mRNA delivery has been shown to enhance CD8^+^ T-cell infiltration into tumors, remodel the immunosuppressive tumor microenvironment, and restore antitumor immune response (Lin et al., 2021; Hu et al., 2025).

### 2.3 Protein and immune checkpoint-related targets

Compared with genes and transcripts, protein targets are more closely linked to functional biological endpoints and can reflect the actual expression and potential functional state of immune checkpoint molecules. PD-L1 constitutes a central therapeutic target and remains the most widely used protein target for identifying tumors with pre-existing immune activation. The predictive value of PD-L1 appears to be both tumor-type- and cellular-compartment-dependent, with higher tumor-cell expression in NSCLC than in RCC or melanoma, whereas in melanoma, PD-L1 expression on tumor-infiltrating immune cells is more strongly associated with clinical outcomes than tumor-cell PD-L1 (Kluger et al., 2017). CTLA-4 and LAG-3 are key inhibitory immune checkpoint receptors that regulate distinct stages of T-cell activation and antitumor immunity. CTLA-4 predominantly restrains the priming and expansion of T cells, whereas LAG-3 suppresses T-cell activation and effector function and cooperates with PD-1 to promote tumor immune escape (Wei et al., 2017; Woo et al., 2012). CTLA-4 and LAG-3 may serve as complementary targets for overcoming resistance to PD-1/PD-L1 blockade. Multiple clinical studies have demonstrated the efficacy of combined CTLA-4 and PD-1/PD-L1 inhibition across several tumor types, including melanoma, metastatic colorectal cancer, renal cell carcinoma, and NSCLC (André et al., 2025; Wolchok et al., 2025; Long et al., 2025; Motzer et al., 2018; Paz-Ares et al., 2021). In contrast, the most definitive evidence for LAG-3 blockade currently comes from advanced melanoma, in which its addition to PD-1 inhibition has improved clinical outcomes compared with PD-1 monotherapy (Tawbi et al., 2022). As a key immune checkpoint involved in T-cell exhaustion and suppression of effector T-cell function, LAG-3 blockade can enhance intratumoral CD8^+^ T-cell responses, whereas its combination with PD-1 inhibition may restore antitumor immunity by relieving nonredundant inhibitory signals and has demonstrated preliminary clinical activity in recurrent glioblastoma and anti-PD-1–refractory classical Hodgkin lymphoma (Armand et al., 2025; Lim et al., 2026).

### 2.4 Metabolite- and metabolism-related pathway determinants

The small molecules produced, transformed, or consumed during cellular metabolic processes indicate whether the microenvironment is in an immunosuppressive metabolic state. The adenosine pathway is primarily characterized by CD39^−^and CD73-mediated adenosine production and downstream immunosuppressive signaling through A2AR and A2BR, which broadly suppresses both innate and adaptive antitumor immunity. Accordingly, CD39, CD73, A2AR, and A2BR have emerged as promising therapeutic targets for improving responses to cancer immunotherapy. Early clinical studies have shown that CD73 blockade combined with PD-L1 inhibition has clinical activity in unresectable stage III non-small-cell lung cancer (Aggarwal et al., 2025). In addition, among patients with metastatic melanoma treated with nivolumab or pembrolizumab, elevated pretreatment serum CD73 activity was associated with a lack of clinical response and shorter progression-free and overall survival. These findings suggest that soluble CD73 may serve as a minimally invasive prognostic biomarker and may also help predict resistance to PD-1 blockade (Turiello et al., 2020). A2A receptor blockade, either alone or in combination with PD-L1 inhibition, has also demonstrated clinical activity in treatment-refractory renal cell carcinoma (Fong et al., 2020). The tryptophan–kynurenine pathway represents another important immunosuppressive axis. A high serum kynurenine/tryptophan (kyn/trp) ratio has been associated with poor outcomes during PD-1 blockade in several cancers, including metastatic NSCLC, renal cell carcinoma, HNSCC (Botti et al., 2020). Additional pathways include the lactate–LDHA–MCT axis and the arginine–ARG1/ARG2 axis (Yu et al., 2024; Peyraud et al., 2022). Although these pathways are supported by strong mechanistic and preclinical evidence, direct clinical proof that their inhibition enhances PD-1/PD-L1 therapy remains limited.

## 3 Mechanistic basis of pan-cancer immunotherapy resistance-associated biomarkers

Biomarkers associated with immunotherapy resistance should not be viewed as isolated biological alterations. Rather, they arise from the interplay among tumor cells, immune cells, stromal components, and metabolic and inflammatory networks. In pan-cancer immunotherapy resistance, the significance of these biomarkers goes beyond indicating changes in a particular molecule, cell population, or metabolic state. More importantly, they reflect key mechanistic states involved in tumor immune escape. Therefore, understanding how resistance-associated biomarkers are generated provides the basis for using them in treatment response prediction, dynamic monitoring, therapeutic stratification, and the rational design of combination therapies (Figure 2).

**FIGURE 2:**
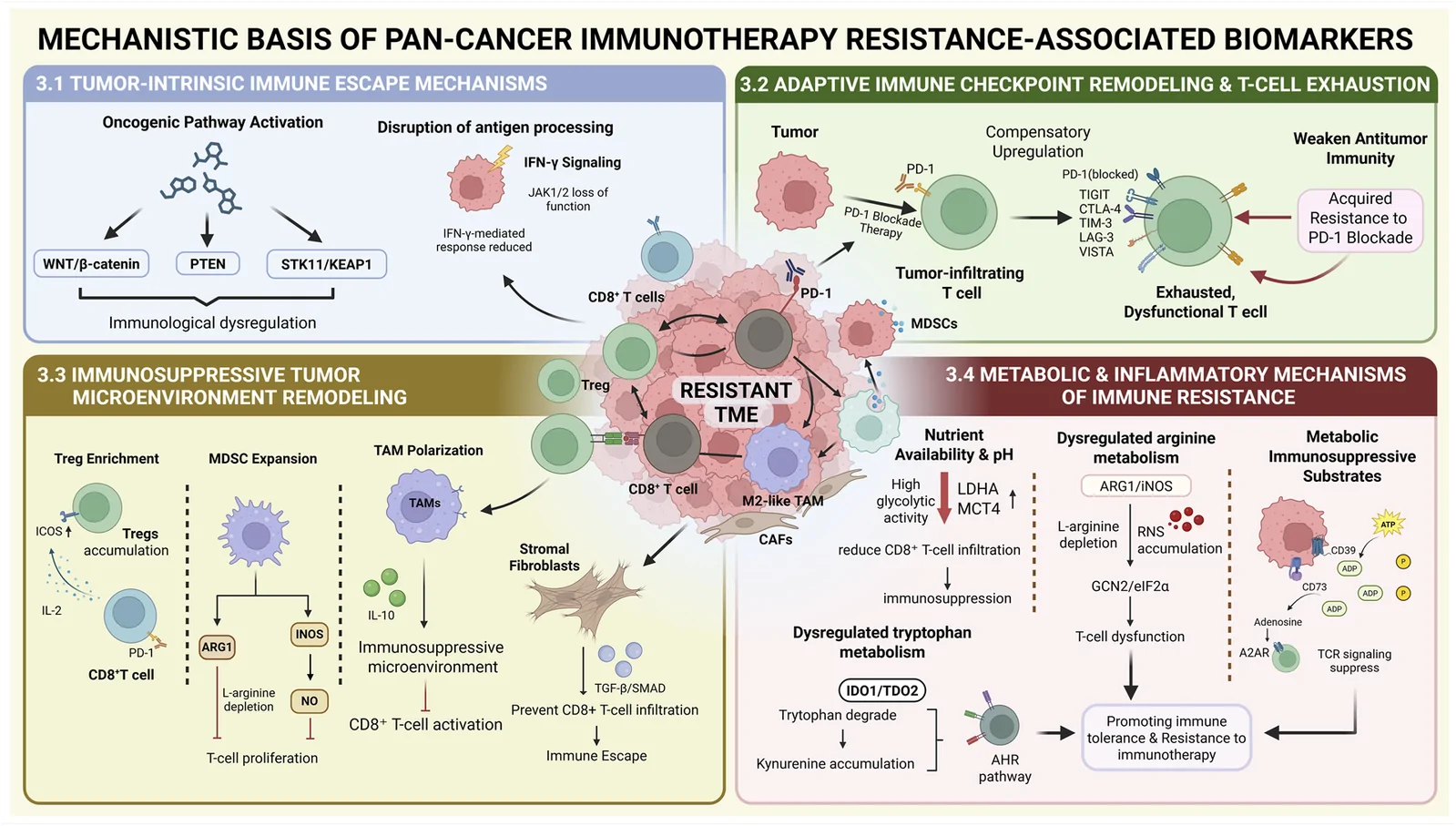
Schematic overview of the major biological mechanisms underlying pan-cancer resistance to immune checkpoint blockade, including tumor- intrinsic alterations, adaptive immune checkpoint regulation, tumor microenvironment remodeling, and metabolic reprogramming.

### 3.1 Tumor-intrinsic immune escape mechanisms

Tumor-intrinsic immune escape represents an important basis for primary resistance to immunotherapy across cancer types. This process is largely driven by genetic alterations and aberrant signaling within tumor cells, which collectively reduce the ability of the immune system to recognize, attack, and eliminate malignant cells. As a result, some tumors remain poorly recognized and insufficiently cleared by T cells, even after checkpoint blockade therapy. One major mechanism is the disruption of antigen processing and presentation. HLA class I molecules on the surface of tumor cells are essential for presenting intracellularly derived tumor antigen peptides to CD8^+^ cytotoxic T lymphocytes. By performing HLA allele-specific copy-number analysis, McGranahan et al. identified HLA allelic imbalance and loss of heterozygosity in NSCLC and showed that HLA loss of heterozygosity (LOH) was associated with enrichment of subclonal neoantigens predicted to bind the lost alleles, suggesting that it may restrict the neoantigen repertoire available for HLA class I presentation (McGranahan et al., 2017). Loss of B2M expression abolished cell-surface MHC class I expression and mediated resistance to CD8^+^ T-cell recognition and killing, whereas subclonal impairment of JAK1 signaling reduced IFN-γ-induced MHC class II expression and conferred resistance to CD4^+^ T-cell-mediated killing in a subset of tumor cells, together contributing to a pan-T-cell immune-escape phenotype (Nielsen et al., 2022). Under normal conditions, IFN-γ serves as a key regulator of the antigen-presentation pathway by activating JAK1/2–STAT1 signaling and upregulating HLA-I and components of the antigen-processing machinery, thereby enhancing HLA-I-mediated antigen presentation (Garcia-Diaz et al., 2017). However, loss-of-function alterations in key IFN-γ signaling components, such as JAK1 or JAK2, can impair or abolish tumor-cell responses to IFN-γ, including the induction of antigen-presentation and chemokine-related genes, thereby potentially contributing to resistance to immune checkpoint blockade (Grasso et al., 2021). Aberrant activation of tumor-intrinsic oncogenic pathways can also drive immune-resistant phenotypes. Aberrant activation of tumor-intrinsic WNT/β-catenin signaling in melanoma cells suppresses CCL4 expression through ATF3, thereby impairing the recruitment of CD103^+^ dendritic cells and the priming of tumor antigen-specific T cells, ultimately leading to T-cell exclusion and resistance to immune checkpoint blockade (Spranger et al., 2015). Tumor-intrinsic PTEN loss can remodel the melanoma immune microenvironment by increasing the production of immunosuppressive factors, including VEGF and CCL2, thereby impairing effector T-cell recruitment and diminishing local antitumor immune activity (Peng et al., 2016). Tumor-intrinsic inactivation of STK11 or KEAP1 can establish an immunologically dysregulated state characterized by the enrichment of immunosuppressive myeloid cells, depletion of cytotoxic CD8^+^ T cells, and relative preservation of effector CD4^+^ T-cell subsets, thereby limiting the efficacy of PD-(L)1 blockade alone (Skoulidis et al., 2024).

### 3.2 Adaptive immune checkpoint remodeling and T-cell exhaustion

Adaptive immunity profoundly shapes the immune landscape of the tumor microenvironment by regulating the activation, infiltration, and effector functions of tumor-specific T cells, thereby providing a fundamental basis for the efficacy of anticancer immunotherapies, including immune checkpoint blockade. Immunotherapeutic pressure can drive adaptive remodeling of the immune checkpoint network within the NSCLC tumor microenvironment, promoting the co-expression of inhibitory molecules such as PD-1, TIGIT, TIM-3, LAG-3, and VISTA on tumor-infiltrating T cells and thereby contributing to acquired resistance to PD-1 blockade alone (Hiltbrunner et al., 2023). Acquired resistance to PD-1 blockade may be mediated by adaptive remodeling of the inhibitory receptor landscape on T cells, characterized by treatment-induced compensatory upregulation of alternative co-inhibitory receptors such as TIM-3, LAG-3, and TIGIT, thereby sustaining T-cell exhaustion and effector dysfunction despite continued inhibition of the PD-1 pathway. Increased expression or co-expression of LAG-3, TIM-3, VISTA, and CTLA-4 may reinforce T-cell dysfunction and exhaustion, thereby weakening antitumor immunity (Kakavand et al., 2017; Koyama et al., 2016; Huang et al., 2017). Hypoxia in the tumor microenvironment can synergize with persistent antigenic stimulation to impair the metabolic adaptability of CD8^+^ T cells, inducing mitochondrial dysfunction and reactive oxygen species accumulation through Blimp-1–mediated repression of PGC-1α, thereby driving T cells toward terminal exhaustion and weakening adaptive antitumor immunity (Scharp et al., 2021).

### 3.3 Immunosuppressive tumor microenvironment remodeling

The immunosuppressive tumor microenvironment continuously undermines antitumor immunity by restricting effector T-cell infiltration and activation, promoting T-cell exhaustion, and enhancing the suppressive functions of regulatory T cells and other immunosuppressive populations, thereby serving as a major driver of both primary and acquired resistance to immunotherapy. Following CD8^+^ T-cell activation by PD-1 blockade, IL-2/ICOS signaling can promote the survival and accumulation of intratumoral Treg cells, establishing a feedback-driven immunosuppressive microenvironment that constrains effector T-cell function, thereby attenuating therapeutic responses and promoting resistance to immunotherapy (Geels et al., 2024). Enrichment of regulatory T cells is a major contributor to this state. Through CTLA-4-dependent suppression of antigen-presenting-cell costimulation, limit the availability of IL-2, and other dependent immunoregulatory mechanisms, tumor-infiltrating Tregs can restrict the activation, expansion, and cytotoxic function of CD8^+^ T cells (Geels et al., 2024; Tekguc et al., 2021; Noyes et al., 2022). Treg accumulation may therefore weaken antitumor immune responses and limit the efficacy of immune checkpoint blockade. Enrichment of tumor-infiltrating Treg cells characterized by FOXP3 and IL2RA expression, together with increased expression of immunoregulatory molecules including OX40, CTLA4, and GITR, is associated with enhanced suppressive activity of Treg cells (De Simone et al., 2016). MDSCs expansion is another key mechanism through which the immunosuppressive tumor microenvironment impairs T-cell effector function (Cartwright et al., 2021; Rodriguez et al., 2007). Research demonstrates that tumor-infiltrating MDSCs contribute to the formation of an immunosuppressive tumor microenvironment by producing nitric oxide (NO) through inducible nitric oxide synthase (iNOS), which induces DNA damage and activates p53-mediated stress responses in CD8^+^ T cells, thereby impairing T-cell proliferation and survival and facilitating tumor immune escape (Cartwright et al., 2021). Tumor-infiltrating MDSCs promote L-arginine (L-Arg) depletion through the upregulation of ARG1, which activates the amino acid starvation response-associated GCN2–eIF2α pathway in T cells, suppresses the expression of Cyclin D3 and CDK4, and consequently induces G0/G1 cell-cycle arrest and impairs CD8^+^ T-cell proliferative capacity (Rodriguez et al., 2007). Tumor-associated macrophages (TAMs) restrict the migration and infiltration of CD8^+^ T cells into tumor regions, and depletion of TAMs can relieve this immunosuppressive barrier, thereby enhancing the therapeutic efficacy of anti-PD-1 immunotherapy (Peranzoni et al., 2018). In addition, TAMs contribute to an immunosuppressive tumor microenvironment by secreting the immunosuppressive cytokine IL-10, which suppresses IL-12 production by tumor-infiltrating dendritic cells (DCs), thereby attenuating IL-12-mediated CD8^+^ T-cell activation and cytotoxic responses. Targeting the CSF-1/CSF-1R axis to deplete TAMs or blocking IL-10/IL-10 R signaling can disrupt this immunosuppressive network, enhance DC-derived IL-12 production and enhance CD8^+^ T cells dependent antitumor immunity, and improve the efficacy of chemotherapy (Ruffell et al., 2014). The efficacy of anti–PD-L1 immunotherapy is jointly influenced by tumor mutational burden (TMB) and a TGF-β-driven immune-excluded microenvironment. Aberrant activation of TGF-β/SMAD signaling in tumor-associated stromal fibroblasts promotes the formation of a fibrotic barrier that restricts CD8^+^ T-cell infiltration into the tumor core, thereby facilitating immune escape (Mariathasan et al., 2018). Meanwhile, although higher TMB is generally associated with improved responses to anti–PD-1/PD-L1 therapy, both its prevalence and predictive performance vary substantially across tumor types, indicating that TMB should be interpreted in a tumor-specific context rather than as a uniformly predictive pan-cancer biomarker (Valero et al., 2021).

### 3.4 Metabolic and inflammatory mechanisms of immune resistance

Tumor metabolic reprogramming and chronic inflammatory signaling provide an important link between adaptive tumor cell survival and resistance to immunotherapy. These mechanisms impair the effector functions of T cells and NK cells mainly by altering nutrient availability, local pH, immunometabolic substrates, and the strength of inflammatory signaling within the tumor microenvironment. Tumor cells competitively deplete glucose in the tumor microenvironment by enhancing glycolysis, resulting in impaired mTOR signaling, glycolytic metabolism, and IFN-γ effector function in T cells, thereby promoting immune evasion (Chang et al., 2015). Tumor-derived LDHA-driven lactate accumulation induces acidification of the tumor microenvironment, impairing T cell and NK cell function by disrupting intracellular homeostasis and suppressing NFAT activation, thereby reducing IFN-γ production and weakening antitumor immune surveillance (Brand et al., 2016). Enhanced glycolytic activity in tumor cells promotes lactate efflux and extracellular acidification through upregulation of the lactate transporter MCT4, thereby reshaping an immunosuppressive tumor niche, reducing CD8^+^ T-cell infiltration and antitumor effector functions, and ultimately driving immune checkpoint therapy resistance. Targeting MCT4 to disrupt glycolysis-dependent lactate metabolism alleviates immunosuppression and restores sensitivity to anti-PD-1 therapy (Yu et al., 2024). Activation of the adenosine pathway promotes the conversion of extracellular ATP into adenosine through CD39 and CD73. Adenosine then acts through the A2A receptor to suppress TCR signaling, cytokine release, and NK-cell effector function (Xia et al., 2023). Tryptophan can serve as a regulatory node in immune metabolism. Tumors degrade tryptophan into Kyn via IDO1 or TDO2, simultaneously causing local tryptophan depletion and Kyn accumulation; these two processes synergistically activate the AHR pathway, promoting immune tolerance and resistance to immunotherapy (Campesato et al., 2020; Solvay et al., 2023). Tumor metabolic reprogramming induces ARG1/iNOS-dependent L-arginine depletion and RNS accumulation in MDSCs, establishing an immunosuppressive microenvironment through GCN2/eIF2α activation and T-cell dysfunction, while metabolic targeting restores antitumor immunity and enhances immunotherapy responses (Raber et al., 2012; Fletcher et al., 2015; De Sanctis et al., 2022).

## 4 Emerging technologies reveal TME heterogeneity

Recent advances in single-cell sequencing, spatial transcriptomics, and highly multiplexed tissue imaging have enabled a more detailed characterization of tumor microenvironmental heterogeneity associated with immunotherapy response and resistance. The integration of scRNA-seq with scTCR-seq allows T-cell functional states to be linked to their clonal lineages. At the single-cell level, response to PD-1 based therapy in NSCLC was associated with the expansion and replenishment of precursor exhausted T cells, whereas nonresponsive tumors failed to achieve a comparable accumulation of this population (Liu et al., 2022). Spatial transcriptomics further reveal heterogeneity in immune-cell localization and intercellular organization. In colorectal cancer, Visium HD identified transcriptionally distinct macrophage subpopulations occupying different spatial niches and localized clonally expanded T cells near macrophages with potential antitumor features (Oliveira et al., 2025). Although this study did not involve an immunotherapy-treated cohort, it demonstrates how high-resolution spatial transcriptomics can resolve immune niches and cellular interactions potentially relevant to treatment response. Together, these findings illustrate how emerging technologies can identify cellular, clonal, and spatial features of the tumor microenvironment that distinguish effective antitumor immunity from resistant states.

## 5 Conclusion

Immunotherapy resistance is a multifactorial and dynamically evolving process driven by tumor-intrinsic alterations, adaptive immune checkpoint remodeling, immunosuppressive cellular networks, and metabolic and inflammatory changes within the tumor microenvironment. Resistance-associated biomarkers can therefore reflect different biological levels, including transcriptional regulation, genomic alterations, protein and immune checkpoint expression, and immunosuppressive metabolic pathways. These biomarkers not only help characterize the mechanisms underlying primary or acquired resistance but may also reveal therapeutically actionable vulnerabilities that can be targeted to restore sensitivity to immune checkpoint blockade. Nevertheless, their levels of clinical validation remain highly heterogeneous. Moreover, shared biological mechanisms do not necessarily translate into uniform biomarker performance across malignancies. The predictive value of a biomarker may depend on tumor lineage, molecular subtype, disease stage, treatment regimen, assay platform, and biomarker threshold. Therefore, future studies should distinguish treatment-specific predictive effects from general prognostic associations and validate resistance-associated biomarkers within clearly defined cancer-specific clinical contexts.
